# Longitudinal Mercury Monitoring within the Japanese and Korean Communities (United States): Implications for Exposure Determination and Public Health Protection

**DOI:** 10.1289/ehp.0900801

**Published:** 2009-07-31

**Authors:** Ami Tsuchiya, Thomas A. Hinners, Finn Krogstad, Jim W. White, Thomas M. Burbacher, Elaine M. Faustman, Koenraad Mariën

**Affiliations:** 1 Department of Environmental and Occupational Health Services and; 2 Institute for Risk Analysis and Risk Communication, University of Washington, Seattle, Washington, USA; 3 National Exposure Research Laboratory, U.S. Environmental Protection Agency, Las Vegas, Nevada, USA; 4 Washington State Department of Health, Olympia, Washington, USA

**Keywords:** consumption, exposure, fish, fish advisories, hair, mercury, longitudinal, reference dose, temporal

## Abstract

**Background:**

Estimates of exposure to toxicants are predominantly obtained from single time-point data. Fish consumption guidance based on these data may be incomplete, as recommendations are unlikely to consider impact from factors such as intraindividual variability, seasonal differences in consumption behavior, and species consumed.

**Objectives/methods:**

We studied populations of Korean (*n* = 108) and Japanese (*n* = 106) women living in the Puget Sound area in Washington State to estimate mercury exposure based on fish intake and hair Hg levels at two and three time points, respectively. Our goals were to examine changes in hair Hg levels, fish intake behavior, and Hg body burden over time; and to determine if data from multiple time points could improve guidance.

**Results/conclusion:**

More than 50 fish species were consumed, with eight species representing approximately three-fourths of fish consumed by the Japanese and 10 species representing approximately four-fifths of fish intake by the Koreans. Fish species responsible for most Hg intake did not change over time; < 10 species accounted for most of the Hg body burden in each population. Longitudinal variability of hair Hg levels changed slowly across the study period. Japanese with hair Hg levels > 1.2 ppm (mean, 2.2 ppm) consumed approximately 150% more fish than those with levels ≤ 1.2 ppm (mean, 0.7 ppm). However, because many participants consumed substantial amounts of fish while having hair-Hg levels ≤ 1.2 ppm, the nutritional benefits offered from fish consumption should be obtainable without exceeding the RfD. We observed a 100% difference in fish intake between open-ended and 2-week recall fish consumption surveys. Open-ended survey data better represent Hg intake as determined from hair Hg levels. Single time-point fish intake data appear to be adequate for deriving guidance, but caution is warranted, as study is required to determine the significance of the different outcomes observed using the two survey time frames.

Mercury, specifically methylmercury (MeHg), has been shown to cause developmental and neurologic effects, with the severest effects being observed after catastrophic exposure to communities in Japan and Iraq [Bakir et al. 1973; Harada 1995; Kondo 2000; National Research Council (NRC) 2000]. Exposure to MeHg can come from many routes, but the most prominent nonoccupational pathway is fish consumption (NRC 2000). For the purpose of public health protection, the U.S. Environmental Protection Agency (EPA) has established a reference dose (RfD) for MeHg of 0.1 μg/kg/day (NRC 2000; U.S. EPA 2000). A hair Hg level of 1.2 ppm is considered to be the exposure equivalent of the RfD (NRC 2000).

Two large, ongoing studies in the Faroe Islands and Seychelles Islands continue to provide insight into the relationship between exposure and effects from Hg (Axtell et al. 2000; Davidson et al. 1995, 2006; Grandjean et al. 1992; 1994; 1997; 1998; Marsh et al. 1987; Myers et al. 1995; 2003; 2007; Steurwald et al. 2000; Weihe et al. 2004; van Wijngaarden et al. 2006). In our smaller study of Japanese and Korean populations, we found that they have distinctly different fish consumption behaviors that greatly impact Hg body burden levels between the two communities (Tsuchiya et al. 2008a, 2008b).

Data from these studies and others have been used by health agencies to establish protective health guidance for fish-consuming populations and communities. To date, exposure data have centered around single time points, with little information available on the temporal aspects of Hg body burden levels or consumption behavior patterns. Multiple sampling of individuals over time may capture longitudinal variability such as intraindividual variability, seasonal variations in consumption behavior, and differences in species type consumed due to seafood availability. Information about temporal variability may impact appropriate public health guidance. As part of the Arsenic Mercury Intake Biometric Study (AMIBS), we examined longitudinal data for hair Hg levels, fish consumption behavior, and estimated Hg exposure within the Korean and Japanese populations at two and three time points, respectively. Hg exposure in these two populations is important because both consume substantially more fish than the national average (Tsuchiya et al. 2008a, 2008b). The main goals of this work were 2-fold: to examine changes in hair Hg levels, fish intake behavior, and Hg body burden over time in these two populations (Japanese and Korean women living in the Puget Sound area of the United States); and to determine if data from multiple time points could improve public health guidance. Survey tools using two different recall time periods were used during this study, allowing us to compare fish intake results with hair Hg levels to determine which survey period provided data best reflecting hair Hg levels.

## Methods

### AMIBS and study population

Detailed descriptions of AMIBS have been published (Tsuchiya et al. 2008a, 2008b). The study included women of childbearing age (18–45 years of age) who identified themselves as Korean, Japanese, or of Japanese or Korean descent (hereafter referred to as Japanese and Korean) and who had lived in the Puget Sound area of Washington State, USA, for at least 6 months. The Japanese cohort (*n* = 106) was interviewed three times and the Korean cohort (*n* = 108) twice across a 14-month period. On the first, second, and third visits, data were obtained for 106, 90, and 85 Japanese individuals, respectively. From the Korean cohort, 108 individuals participated on the first visit, with 63 returning a second time. Informed consent was obtained from all study participants, and the study design and materials were approved by the State of Washington Department of Social and Health Services Human Research Review Board.

### AMIBS temporal data gathering

The population and sample collection have been described previously (Tsuchiya et al. 2008b). Enrollees completed an oral fish consumption survey (FCS), provided biological samples based on their consent (hair, urine, blood, and/or toenails), had their toenails marked and hair sample location recorded for determining growth rates at a later date, and completed a self-administered food-frequency questionnaire (FFQ).

Participants returning for second and third visits were interviewed in the same manner as during the first visit. The recall time period for the FCS was different between first and second or third visits. For the first visit, the surveys were open-ended, and individuals could respond to questions about their fish consumption behavior spanning a year-long period. For the second and third visits, information obtained from the surveys covered the last 2 weeks prior to the date of the visit; thus, they were 2-week recall surveys. Two time periods for the survey tools were chosen so that *a*) a longer-term average of exposure could be determined that would better correspond to the weighted average of exposure obtained from the hair sample collected, and *b*) recall bias could be better controlled using a short time frame, although quantification of consumption of the less frequently consumed species would be compromised. A 2-week recall period instead of a 1-month time frame was chosen to reduce the impact of the respondent’s memory while still providing a time period sufficiently long during which participants would likely consume multiple fish meals.

The FFQ was a validated tool for obtaining dietary information and was augmented for use in this study through the addition of an accompanying section specific to food items consumed by these two populations (Kolar et al. 2005; Kristal et al. 1994; Patterson et al. 1999). The FCS was based on surveys previously used with several Native American tribes, recreational fishers, Asian Pacific Islanders, and the general public (Landolt et al. 1985; Mariën and Patrick 2001; Toy et al. 1996).

In the present study we used the hair Hg values obtained from all visits to the clinics and data from the open-ended and the 2-week recall survey tools.

### Hair sampling, fish collection, and Hg analysis

As previously described (Tsuchiya et al. 2008b), multistrand proximal hair segments approximately 6 cm long and 0.5 cm in diameter were cut from the nape of the neck and were analyzed with a detection limit near 0.01 ng Hg (corresponding to 0.001 ng/mg for 10 mg of hair). Hair Hg quality assurance included the measurement of certified reference materials before and after sample analyses plus participation in the Health Canada Mercury in Hair Interlaboratory Comparison Program (Gill 2004).

Fish commonly consumed by the Japanese and Korean communities in the Puget Sound area were purchased at local Asian grocery stores. We determined total Hg in tissue using cold-vapor atomic absorption with a typical instrumental detection limit of 0.2 ng/mL. Choice of fish species and tissue Hg values used were described previously (Tsuchiya et al. 2008a, 2008b).

### Statistical analyses

We determined the extent of Hg exposure for each individual by estimating Hg intake based on fish consumption data and by analyzing hair for Hg (Tsuchiya et al. 2008b). Paired *t*-tests were performed on the estimated fish intake levels and hair Hg levels to compare the returning Japanese and Korean participants over time. In addition, we performed Student’s *t*-tests to compare returning participants with those who did not return in order to determine if the returning participants represented the total study population. Levene’s test was performed to check for homogeneity of variance across hair Hg levels and fish intake levels obtained from the multiple enrollee visits over the study period. When equal variances could not be assumed, significance was based on the assumption of unequal variances. Estimated Hg intake results from the FCS as well as hair Hg levels were compared with the RfD on a population-specific basis. Statistical analyses were performed using a significance level of 5% (*p* < 0.05) and completed using Stata (StataCorp, College Station, TX), Excel (Microsoft Corporation, Redmond, WA), and SPSS (SPSS Inc., Chicago, IL).

## Results

### Japanese: fish consumed and Hg intake over time

Of the 106 Japanese women initially enrolled in the study (first visit), 90 and 85 returned for a second and third visit, respectively. The mean estimated total fish intake values were significantly different between the first visit and the latter two visits, with the 2-week time frame mean intake values being approximately half of the open-ended survey value (60 g/day vs. 34 and 31 g/day for first vs. second and third visits) (Table 1). Mirroring these results, the mean estimated individual Hg intake of 7.2 μg/day for the first visit fell to 2.7 and 2.5 μg/day, respectively, for the second and third visits (Table 2).

Table 1 shows the fish species most consumed by the Japanese cohort during the study period and indicates the percentage that each species represents of the total amount consumed. The list of species most consumed did not change considerably over the course of the study, as eight species represented between two-thirds and three-fourths of the total amount of fish consumed. Of those eight species, salmon, mackerel, and light tuna accounted for approximately half of the total. No significant difference in fish consumption level was observed between the 85 individuals present for all visits and the 21 participants who did not return. This suggests that fish intake levels of the returning enrollees represent those of the whole population; accordingly, first-visit data are provided for the total sample only in Tables 1 and 2.

The fish species that contributed most to Hg body burden did not vary greatly throughout the course of the study period (Table 2). Species responsible for > 5% of the total Hg intake are listed, and they represent approximately 60–90% of the estimated Hg intake within this population over the course of the study.

### Korean: fish consumed and Hg intake over time

Of the 108 Korean participants who completed the first visit, 63 returned for a second visit. Table 3 lists the 10 most highly consumed fish species as reported by the participants at the first and second visits. Consumption data for the first visit are presented separately for the entire Korean cohort and for those who returned for a second visit, because there was a significant difference in total fish intake levels between those who returned for a second visit and those who did not. Although this suggests that the total intake of the returning participants may not reflect the population as a whole, the percentage that each species contributed to the total intake was very similar during the first visit between the whole population and those 63 individuals who returned for a second visit. Over the course of the two visiting periods, 10 species represented > 4 g of every 5 g of fish consumed, with squid, mackerel, yellow croaker, and salmon representing more than half of the total intake across the study period.

The eight species listed in Table 4) represent approximately 65–80% of the estimated Hg intake across the two visits covering the study period. Nearly 50% of the estimated Hg intake at the second visit came from three species [white and light tuna (both canned) and flounder/sole]. At the second visit, participants reported consuming more than twice as much light tuna (canned) than at the first visit, resulting in a doubling of the percentage of Hg intake from this species to 17% at the time of the second visit. Both times the survey was conducted, white (albacore) tuna (canned) was among the species providing the highest percentage of total Hg intake but was not one of the 10 most highly consumed species.

Average estimated total fish intake values were examined between visits (Table 3). Intakes between the first and second visits were significantly different, with the first-visit whole sample of 108 individuals and the subsample of 63 individuals having intake values of 59 g/day and 72 g/day, respectively. These first-visit samples using open-ended survey results were approximately twice the second visit average consumption value (29 g/day) obtained using the 2-week time frame survey results (Table 4). Similar results were observed with the average estimated Hg intake values (Table 4). The first-visit values for the whole sample and the subsample returning for a second visit were 5.3 and 5.1 μg/day, respectively. The second visit results yielded an intake of 2.6 μg/day. These decreases are similar to those observed in the Japanese population.

### Japanese: longitudinal fish intake and hair Hg levels

We analyzed fish consumption rates and hair Hg levels of the total population across visits. Further, we compared those parameters in the subpopulation considered overexposed (i.e., hair Hg > 1.2 ppm) with the group that was not (hair Hg ≤ 1.2 ppm) (Table 5). Mean fish intake reported at the first visit was approximately 60 g/day for all participants and 64 g/day for the 85 participants who returned for both additional visits (Tsuchiya et al. 2008a). The hair Hg levels were 1.6 ppm and 1.7 ppm for the population and the returning enrollees, respectively. No significant difference was found between the two samples, suggesting that hair Hg values from returning participants represent those of the entire population (Table 5).

Compared with the first visit when the open-ended survey was used, total reported fish intake for the 85 returning enrollees was significantly decreased at the second and third visits (Table 5). No significant difference was observed between the second and third visits when the recall period for the survey was limited to 2 weeks. Total mean hair Hg levels showed a difference (*p* < 0.05) across all visits, suggesting that there is a decrease in hair Hg levels over time along with an observed decrease in the range of the 95% confidence interval (CI) over time (Table 5).

At each visit, the group with hair Hg levels > 1.2 ppm had an average fish daily consumption rate 1.5 to 2 times greater than the group with hair Hg levels ≤ 1.2 ppm (Table 5). This suggests that the overexposed group does consume more fish. Differences in intakes were significant at each visit with the exception of the second visit, where those overexposed women consumed approximately 38 g/day compared with 27 g/day for those who were not overexposed. For each visit and for each sample size, the differences in hair Hg levels in overexposed women were approximately 3 times greater than those in women who were not overexposed (*p*< 0.05).

### Korean: longitudinal fish intake and hair Hg levels

Within the Korean cohort, the fish consumption rates and hair Hg levels were analyzed across the two visits, including a comparison of the groups considered overexposed (i.e., those with hair Hg > 1.2 ppm) with those who were not (hair Hg ≤ 1.2 ppm). Fish intake level from the first visit for all participants was approximately 60 g/day, whereas for the 63 returning women, the intake was higher (*p* < 0.05) at 72 g/day (Tsuchiya et al. 2008a). The hair Hg levels at the first visit were identical (0.8 ppm). As with the Japanese cohort, the hair Hg samples of the returning Korean participants (*p* < 0.05) do suggest that the returning participants represent the population as a whole (Table 5).

Between the first and second visits, the fish intake levels diminished significantly from 60 g/day and 72 g/day for the whole population and the subsample of 63 that returned, respectively, to 29 g/day for the subsample at the second visit. Total mean hair Hg levels were different (*p* < 0.05) between the two visits, with mean values rising from 0.8 ppm to 0.9 ppm.

Individual fish intake data for the two visits were separated by those who had hair Hg levels ≤ 1.2 ppm versus those > 1.2 ppm (Table 5). We found no significant difference between those who were overexposed and those who were not overexposed during the first visit for either the whole cohort (63 vs. 59 g/day, respectively) or the returning participants (66 vs. 73 g/day, respectively). One difference between the whole cohort and the returning participants during the first visit is that the overexposed group within the returning participants consumed less fish than those who were not overexposed (66 vs. 73 g/day). However, the significance of this outcome is unclear, as only a small percentage (< 15%) from either the whole cohort or the returning participants was in the overexposed group. On the second visit, using the 2-week recall time period for the survey, the overexposed returning participants consumed more fish than those who were not overexposed (*p* < 0.05). Hair Hg levels in overexposed individuals were approximately 2.5 to 3 times higher (*p* < 0.05) than in those who were not overexposed at each of the two visits, suggesting that the Hg body burden of the overexposed group was greater than for the not overexposed group.

### Japanese: temporal hair Hg variability

**Figure 1:**
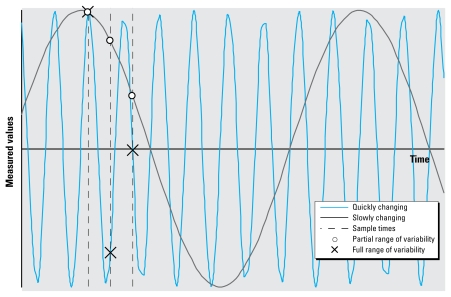
Illustration of hypothetical longitudinal sampling capturing temporal variance otherwise missed by single time-point sampling. Multiple sampling could capture the full range of the process variability missed by sampling at a single time. A process that changes rapidly (blue curve) compared with the sampling frequency (dashed lines) can produce sample values that represent the full range of the process variability. When the values change slowly (black curve) compared with the sampling frequency, as was the case with the Japanese population, the samples show only part of the total variation.

The longitudinal sampling strategy enabled us to examine the variability of hair Hg values over time. For example, sampling at a single time could fail to capture seasonal variations in fish availability or individual fish intake, and thus capture only a portion of the variability. Temporal variance could be measured if the hair Hg values changed rapidly compared with the sampling frequency, such that the Hg levels for each visit over time would provide high and low values as well as those in between (Figure 1, blue curve). If hair Hg values changed slowly compared with the sampling frequency, little variability would be observed across the study period time frame (Figure 1, black curve). This latter occurrence would underrepresent the normal variability.

To determine if this variability could be captured, we conducted repeated sampling. For participants who completed three visits, a temporal plot of each individual’s hair Hg levels did not clearly identify whether the three samples captured the range of variability for each subject (data not shown). The change for each individual over time was generally less than the difference between subjects. This suggests that additional variability might be observed for each participant if more samples were taken. With only three samples, it is difficult to determine if we have sampled the full range of variability for each subject. If sample values gradually rise and fall over time, then these multiple longitudinal samples run the risk of underrepresenting the normal variability observed over a longer time frame. Given that the change over time for each participant was less than the difference between participants at each time point, it is possible that these data from the three clinic visits represent only part of the longitudinal variability that would have been observed had the study period been longer. Accordingly, this suggests that the change over time is slow.

To further examine if the variability changed frequently (fast) or deliberately (slow) with respect to sample timing, we tabulated the number of individual hair Hg levels in which the value of the second visit lies between the values of the first and third visit (Hg_first_ < Hg_second_ < Hg_third_ or Hg_first_ > Hg_second_ > Hg_third_). If variation between subsequent hair Hg levels (Figure 1, blue curve) is sufficiently large, then each will appear to be an independent sampling from the distribution, and each observation in the sequence is equally likely to be the largest, or the smallest, or the one in between. Such a process would be modeled by a binomial distribution, in which the second observation lies between the values of the first and third observation only one-third of the time. Accordingly, we would expect about 28 samples (of the 85 total) to have Hg_second_ values between their Hg_first_ and Hg_third_ values. However, 41 subjects had an Hg_second_ value between their Hg_first_ and Hg_third_ values, suggesting (*p* < 0.05) that the rapidly changing process (Figure 1, blue curve) does not represent the temporal variability and that the actual change in variability may be slow (Figure 1, black curve).

## Discussion

Based on first-visit data, the Japanese and Korean populations in the present study had previously been shown to rely on a few select fish species for the majority of their fish intake (as well as Hg intake) (Tsuchiya et al. 2008b). The fish species most consumed by each population and those contributing the greatest percentage to the total Hg intake changed little over the course of the study. The proportion of black cod, salmon, and halibut consumed did change during the course of the study period; seasonal fluctuations in availability of these species may explain some of this variation in consumption behavior because, for example, the fresh halibut and salmon seasons in the study area do not run year round (www.Dontbuyhalibut.com 2009; Fish Ex 2000; Washington State Department of Fish and Wildlife 2009a, 2009b). Intervention activities may have contributed to the observed decrease in the contribution to the Hg body burden by white (albacore) tuna (canned) in the Japanese population. After the first-visit interviews were completed, individuals were given educational materials and advised to switch to alternative fish species with lower Hg concentrations. The Korean population data provided for less definitive insight, as participants returned but once during the study period. There was a marked and inexplicable increase in the proportion of light tuna (canned) consumed relative to total consumption that resulted in this species being responsible for greater than 10% of the Hg intake during the second visit. This occurred while there was no decrease in the contribution of white (albacore) tuna (canned) to the Hg body burden. Further, the increase in the percentage of Hg intake for light tuna could not be explained by a significant decrease in the proportion of white tuna consumed over time; first and second survey results indicate that white tuna was responsible for only 3.2% and 2.6% of all fish consumed, respectively. In spite of changes within a few species, the majority of fish consumed and Hg body burden obtained from consumption came from the same select species of the > 50 species consumed by these two populations. Accordingly, should guidance be warranted, the species listed (Tables 1–4) can provide the foundation from which to derive public health guidance for these two populations and for others having similar dietary behaviors.

Based on first-visit data, approximately one in two Japanese participants exceeded the RfD, whereas only a small percentage of the Korean population was overexposed (Tsuchiya et al. 2008b). These findings did not change when we included data obtained from multiple time points. However, across the study period, Japanese participants who were overexposed to Hg had 3 times higher hair Hg levels, on average, and consumed fish at a rate 1.5 to 2 times higher than those not overexposed. This is similar to National Health and Nutrition Examination Survey (NHANES; 1999–2004) findings, where blood Hg levels increased with increases in monthly consumption of fish (both finfish and shellfish) (Mahaffey et al. 2009). Similar results were observed for the Korean group in that the overexposed participants had hair Hg levels 2.5 to 3 times higher than those who were not overexposed. For the Japanese and Korean populations, individuals entering the study who were not overexposed consumed 40–60 g/day of fish (first-visit data for all), indicating that fish can be consumed in quantity without exceeding the RfD and in sufficient quantity to offer individuals the nutritional benefits that consuming fish provides.

We examined the temporal variability of hair Hg levels within the Japanese population and suggest that the change is gradual and that the length of this study is too short to accurately define the normal variability of hair Hg levels. The study length may explain the differences observed between these results and those obtained from NHANES, in which multiple regression modeling found differences in blood Hg levels between the data sets obtained across a longer time frame (6 years) (Mahaffey et al. 2009). In addition, for many individuals the hair samples from sequential visits overlapped with respect to exposure period. Specifically, the average time between visits was 4.75 months, but the length of hair samples provided for a weighted average of exposure corresponding to approximately a 5.5- to 6-month period (assuming a hair growth rate of 1–1.1 cm/month). Therefore, exposures contributing to the Hg level in the distal portion of the first hair sample may have also contributed to observed levels in the proximal end of the next sample. If the overlap between survey periods had a large impact, there would have been less likelihood of observing a significant difference between results of the first and second visits than between results of the first and third visits (as the time periods represented by these hair strands do not overlap). However, we observed a statistically significant difference between the first and second visits for both populations and between the first and third visits for the Japanese. Even with the significant differences observed between visit periods, the overlap could have some influence, but it is difficult to quantify the extent to which this variable confounded and impeded the observation of actual longitudinal differences.

The relatively slow change in observed longitudinal variability in hair Hg levels was supported by plotting the individual hair Hg levels of all returning participants based on visitation date over the study period to discern if the population had cyclic variation in hair Hg levels (data not shown). The temporal plot of hair Hg levels did not identify a single time period (month or season) with lower levels, suggesting that the fish that are preferred and that are responsible for maintaining an individual’s Hg body burden can be obtained throughout the year.

The observed decrease in hair Hg values within the Japanese population may represent a portion of the normal variation associated with gradual dietary changes or may have been impacted by attempts to intervene at the first visit through education. A longitudinal sampling protocol of this type may have advantages, but the time period would need to be extended to resemble that used within NHANES. The results presented here suggest that guidance established to provide public health benefit may not be greatly improved by multiple sampling events over a relatively short time period and that single time-point data may be adequate.

Mean fish intake levels based on open-ended recall were about 2 times higher than those based on 2-week recall. To ascertain which recall time period provided intake data that best reflected the hair Hg results, we used a prescriptive comparative approach. For both the Japanese and Korean populations, we determined the fish tissue Hg concentrations representing the contaminant levels in an “average fish” that if consumed daily would yield an exposure level equivalent to the RfD and a hair Hg level of 1.2 ppm. The tissue Hg level of this fish can then be compared with the estimated mean fish tissue Hg concentration of fish consumed by each population at a specific sample time point derived from FCS data. There are limitations to this comparison, because the fish intakes are estimates and the fish tissue Hg levels may not accurately reflect those consumed by an individual or population. However, it is reasonable to assume that an approximation of weighted means of tissue Hg levels based on consumption patterns for the two populations can be obtained to compare with this average fish.

To obtain average fish values, we multiplied the RfD (0.1 μg/kg/day) by mean body weight values for each population (55 and 59 kg for the Japanese and Koreans, respectively) and divided by average intake rates obtained from open-ended time period results (first visit) and 2-week time period results for all returning participants (second/third visits). The Japanese population consumed 60 g/day (first visit, open ended), which yields a tissue Hg level in this average fish of approximately 92 ppb, whereas consuming 33 g/day and 31 g/day (second/third visits, 2-week recall) would yield tissue Hg levels exceeding 160 ppb. For the Korean population, the tissue Hg levels in this average fish would be approximately 100 ppb and exceed 200 ppb for the open-ended and 2-week recall period results, respectively.

To determine a weighted mean fish tissue Hg level representative of intake by the two populations at each sample point, we multiplied the fish species consumption percentages for fish consumed at each sample time point by the contaminant level for each species consumed, and then summed. Open-ended recall period results indicate that the mean tissue Hg levels were approximately 90 ppb and 64 ppb for the Japanese and Korean populations, respectively. When using the 2-week recall period, the mean fish tissue Hg level of consumed fish was approximately 80 and 65 ppb for the Japanese population (second and third visits, respectively). The mean tissue Hg level of Korean participants returning for the second visit was 75 ppb. These mean tissue Hg levels are similar to those obtained from the open-ended average fish tissue Hg levels for the Japanese and Korean populations (90 and 100 ppb, respectively) while being approximately half, or less than half, of the 2-week recall tissue Hg level of average fish that would need to be consumed to attain a hair Hg level of 1.2 ppm.

The Japanese had slightly more overexposed individuals than less-exposed, and their mean hair Hg level (range 1.4–1.6 ppm) was slightly > 1.2 ppm. Therefore, we would have expected the mean tissue Hg concentration to be > 90 ppb because this level is associated with 1.2 ppm Hg in hair. This comparative technique may not have been sufficiently sensitive to observe this difference. Regardless, the open-ended survey results more accurately depict the intake associated with the observed hair Hg levels in the Japanese population. For the Koreans, the average fish tissue Hg level of 100 ppb was much greater than the 64 ppb observed; however, this should be expected, as nearly 90% of the individuals had hair Hg levels < 1.2 ppm (mean range 0.8–0.9 ppm). For these data, the open-ended study time frame provides results that reflect actual hair Hg levels better than the 2-week time frame.

The disparity between the 2-week period fish intake results and actual hair Hg levels may be due to the inability of the short time frame to properly quantify the consumption of fish species other than those consumed frequently or very frequently. One method of possibly addressing this issue may come from an approach such as the one used by Kipnis et al. (2008) in which FFQ data were used to supplement zero values provided in a 24-hr recall survey.

## Conclusion

Multiple samplings across a year-long period support earlier findings that a significant portion of the Japanese population has mercury intake levels exceeding the RfD, whereas the Korean population has only a small percentage of individuals overexposed. However, those individuals not overexposed consume 40–60 g/day of fish, suggesting that fish can be consumed in quantity without exceeding the RfD and in sufficient quantity as to obtain the nutritional benefits that consuming fish provides. Some individuals who consumed substantial amounts of fish did not exceed the RfD. We identified no fundamental problems with using single time-point fish intake data for deriving health guidance. We are concerned that different survey recall time frames may have affected the measured consumption rates, and we conclude that data derived using a short recall time period may produce intake values that do not represent average exposure levels for the population or group studied. This is significant, as many health advisories are based on estimated fish intake and estimated Hg intake data without the benefit of biological sample data. This is the first work that we are aware of that has attempted to compare fish intake from open-ended results with short (2-week) recall time period results. We suggest that caution is warranted, and further study is required to determine the significance of the markedly different outcomes observed using the two survey time frames.

## Figures and Tables

**Table 1:** For Japanese, fish species most consumed (> 4% of total) across three clinic visits.

		Percent of total consumption		
Species	Hg (μg/kg)	Visit 1 (*n* = 106)^a^	Visit 2 (*n* = 90)	Visit 3 (*n* = 85)
Salmon	72	29.0	28.0	36.3
Mackerel	40	9.1	18.1	10.6
Black cod	97	6.5	4.5	2.6
Squid	39	5.6	5.5	3.1
Light tuna (canned)	127	5.0	4.8	8.3
Halibut	216	4.4	0.6	3.3
Ahi	185	4.2	4.3	6.7
Cod	115	1.2	8.7	5.5
Total		65	75	76
Mean individual fish intake (g/day)		59.5	33.7	31.3

The fish species most consumed exceeded 4% of the total during at least one visitation period. Sample sizes reflect total participant number for that visit.

a Data from Tsuchiya et al. (2008b).

**Table 2:** For Japanese, fish species providing greatest Hg intake (> 5% of total) across three clinic visits.

		Percent of total Hg		
Species	Hg (μg/kg)	Visit 1 (*n* = 106)^a^	Visit 2 (*n* = 90)	Visit 3 (*n* = 85)
Salmon	72	17.0	25.0	32.0
White (albacore) tuna (canned)	361	8.7	5.7	2.8
Halibut	216	7.9	1.6	8.7
Ahi	185	6.5	9.7	15.1
Light tuna	127	5.3	7.3	12.8
Black cod	97	5.3	5.3	3.2
Red snapper	221	3.8	5.3	3.4
Mackerel	40	3.0	8.8	5.1
Cod	115	1.1	12.2	7.8
Total		59	81	91
Mean individual estimated Hg intake (μg/day)		7.2	2.7	2.5

The fish species providing the greatest Hg intake exceeded 5% of the total during at least one visitation period. Sample sizes reflect total participant number for that visit.

a Data from Tsuchiya et al. (2008b).

**Table 3:** For Koreans, fish species most consumed (> 4% of total) across two clinic visits.

		Percent of total consumption		
Species	Hg (μg/kg)	Visit 1 (*n* = 108)^a^	Visit 1 (*n* = 63)	Visit 2 (*n* = 63)
Squid	39	23.0	21.2	10.2
Mackerel	40	12.0	13.1	16.4
Yellow croaker	53	11.0	13.1	15.4
Salmon	72	9.1	8.2	7.8
Flounder/sole	147	6.3	6.0	9.1
Light tuna (canned)	127	5.6	4.7	12.2
Black cod	97	4.8	5.3	0.5
Pike mackerel	30	4.3	4.6	5.1
Pollack	22	3.5	4.1	4.4
Ahi	185	2.9	2.3	4.4
Total		83	83	86
Mean individual fish intake g/day		59.1	71.7	29.1

Visit 1 results are shown as total sample (*n* = 108) and sample of enrollees returning for second visit (*n* = 63). The fish species most consumed exceeded 4% of the total during at least one visitation period.

a Data from Tsuchiya et al. (2008b).

**Table 4:** For Koreans, fish species providing greatest Hg intake (> 5% of total) across two clinic visits.

		Percent of total Hg		
Species	Hg (μg/kg)	Visit 1 (*n* = 108)^a^	Visit 1 (*n* = 63)	Visit 2 (*n* = 63)
White (albacore) tuna (canned)	361	14.0	12.7	15.2
Flounder/sole	147	10.0	12.6	14.5
Squid	39	10.0	11.8	4.2
Light tuna (canned)	127	7.9	8.6	17.0
Salmon	72	7.4	8.5	6.1
Yellow croaker	53	6.4	9.9	9.1
Ahi	185	4.9	6.0	9.1
Mackerel	40	4.5	7.5	6.6
Total		65	78	82
Mean individual estimated Hg intake (μg/day)		5.3	5.1	2.6

Visit 1 results are shown as total sample (*n* = 108) and sample of enrollees returning for second visit (*n* = 63). The fish species providing the greatest Hg intake exceeded 5% of the total during at least one visitation period.

a Data from Tsuchiya et al. (2008b).

**Table 5:** Total fish intake and hair Hg levels for each clinic visit for the Korean and Japanese cohorts.

		First visit (all)			First visit			Second visit			Third visit		
		Total	≤ 1.2 ppm	> 1.2 ppm	Total	≤ 1.2 ppm	> 1.2 ppm	Total	≤ 1.2 ppm	> 1.2 ppm	Total	≤ 1.2 ppm	> 1.2 ppm
Japanese	No.	106	50	56	85	36	49	85	40	45	85	41	44
Total fish intake (g/day)	Mean	59.5^a^,^b^	41.5	75.6	63.5^b^	46.0	76.4	33.7^c^	26.6^d^	38.2^d^	31.3^c^	23.0	39.1
	95% CI	50.3–68.8	31.4–51.7	61.9–89.3	52.8–74.2	13.3–59.3	61.5–91.2	26.9–38.6	18.4–34.8	30.1–46.2	26.1–36.6	16.7–29.3	31.5–46.7
Hair Hg (ppm)	Mean	1.6^a^,^e^	0.8	2.3	1.7^e^	0.8	2.4	1.5	0.7	2.2	1.4	0.7	2.1
	95% CI	1.3–1.8	0.7–0.8	1.9–2.7	1.4–2.0	0.7–0.9	2.0–2.8	1.3–1.8	0.6–0.8	1.9–2.6	1.2–1.6	0.6–0.8	1.8–2.7
Korean	No.	108	94	14	63	54	9	63	51	12			
Total fish intake (g/day)	Mean	59.1^a^	58.5^f^	63.3^f^	71.7	72.6^g^	66.2^g^	29.1	25.3	45.3			
	95% CI	47.5–70.7	45.3–71.6	46.7–80.0	54.0–89.3	52.2–93.0	49.3–83.2	23.0–35.2	19.3–31.3	28.0–62.6			
Hair Hg (ppm)	Mean	0.8^a^,^h^	0.6	1.6	0.8^h^	0.6	1.6	0.9	0.7	1.9			
	95% CI	0.7–0.8	0.6–0.7	1.3–1.9	0.7–0.9	0.5–0.7	1.4–1.8	0.7–1.0	0.6–0.7	1.6–2.1			

First-visit data are provided for all enrollees; first, second, and third visit data are provided for those who completed the study.

a Data from Tsuchiya et al. (2008b).

b–h Values with the same letter are not significantly different between visits or between exposure group within visits; remaining values are significant (*p* < 0.05) between visits and between ≤ 1.2 ppm and > 1.2 ppm exposure groups within visits.
